# Nexus Between Spermidine and Floral Organ Identity and Fruit/Seed Set in Tomato

**DOI:** 10.3389/fpls.2019.01033

**Published:** 2019-09-25

**Authors:** Savithri U. Nambeesan, Autar K. Mattoo, Avtar K. Handa

**Affiliations:** ^1^Department of Horticulture, University of Georgia, Athens, GA, United States; ^2^Sustainable Agricultural Systems Laboratory, USDA-ARS, Beltsville Agricultural Research Center, Beltsville, MD, United States; ^3^Center of Plant Biology, Department of Horticulture and Landscape Architecture, Purdue University, West Lafayette, IN, United States

**Keywords:** polyamines, seed set, parthenocarpy, gibberellins, transgenic tomatoes

## Abstract

Polyamines (PAs) constituting putrescine (Put), spermidine (Spd), and spermine (Spm) are ubiquitous in all organisms and play essential roles in the growth and developmental processes in living organisms, including plants. Evidences obtained through genetic, biochemical, and transgenic approaches suggest a tight homeostasis for cellular PA levels. Altered cellular PA homeostasis is associated with abnormal phenotypes. However, the mechanisms involved for these abnormalities are not yet fully understood, nor is it known whether cellular ratios of different polyamines play any role(s) in specific plant processes. We expressed a yeast spermidine synthase gene (*ySpdSyn*) under a constitutive promoter *CaMV35S* in tomato and studied the different phenotypes that developed. The constitutive expression of *ySpdSyn* resulted in variable flower phenotypes in independent transgenic lines, some of which lacked fruit and seed set. Quantification of PA levels in the developing flowers showed that the transgenic plants without fruit and seed set had significantly reduced Spd levels as well as low Spd/Put ratio compared to the transgenic lines with normal fruit and seed set. Transcript levels of *SlDELLA*, *GA-20oxidase-1*, and *GA-3oxidase-2*, which impact gibberellin (GA) metabolism and signaling, were significantly reduced in bud tissue of transgenic lines that lacked fruit and seed set. These findings indicate that PAs, particularly Spd, impact floral organ identity and fruit set in tomato involving GA metabolism and signaling. Furthermore, we suggest that a nexus exists between PA ratios and developmental programs in plants.

## Introduction

Polyamines (PAs), putrescine (Put), spermidine (Spd), spermine (Spm), and thermospermine (Tspm) are hormone-like biogenic amines ubiquitously present in all living organisms including plants. They have been implicated in regulating growth and developmental processes in plants (Kaur-Sawhney et al., 1988; Martin-Tanguy, 2001; Alcazar et al., 2005; Mattoo and Handa, 2008; Nambeesan et al., 2008; Igarashi and Kashiwagi, 2010; Nambeesan et al., 2010; Pegg, 2016; Handa et al., 2018; Tiburcio and Alcázar, 2018). PAs impact primary, lateral and adventitious root development (Flores and Galston, 1982), plant architecture (Cui et al., 2010), *in vitro* plant regeneration by somatic embryogenesis (Bastola and Minocha, 1995) and organogenesis (Ikeuchi et al., 2016), flowering (Malmberg and McIndoo, 1983; Tiburcio et al., 1988), fruit ripening (Mehta et al., 2002), and leaf and flower senescence (Kaur-Sawhney et al., 1980; Nambeesan et al., 2010; Mattoo and Sobieszczuk-Nowicka, 2019). Genetic, biochemical, and transgenic approaches have demonstrated that the metabolism of various PAs is tightly regulated, and imbalance in their homeostasis results in abnormal phenotypes (Kaur-Sawhney et al., 1980; Flores and Galston, 1982; Malmberg and McIndoo, 1983; Kaur-Sawhney et al., 1988; Tiburcio et al., 1989; Bastola and Minocha, 1995; Mehta et al., 2002; Ge et al., 2006; Cui et al., 2010; Nambeesan et al., 2010; Ikeuchi et al., 2016; Murray-Stewart et al., 2018). Furthermore, each PA may have a different effect on metabolism, growth, and development in plants (Handa and Mattoo, 2010; Mattoo et al., 2010; Tiburcio and Alcázar, 2018). Mutations in PA biosynthetic pathway or inhibitors that reduce Put and Spd levels cause plant lethality (Kusano et al., 2008). In addition, it is now apparent that Spm and Tspm play significant role(s) in abiotic responses and xylem differentiation, respectively (Takahashi and Kakehi, 2009; Yoshimoto et al., 2016; Seiff and Shelp, 2019). However, mechanisms that impart PA-associated phenotypes are not yet fully understood.

A close association of PAs with floral development in plants is known (Galston and Sawhney, 1990; Walden et al., 1997). Specifically, PA levels have been correlated with flower development and fruit bearing in olive (Pritsa and Voyiatzis, 2004; Pritsa and Voyiatzis, 2005), apricot (Alburquerque et al., 2006), and plum (DeDios et al., 2006). Early studies also reported higher Put, Spd, and Spm in floral organs of a male-sterile *stamenless-2* (*sl-2/sl-2*) tomato mutant (Rastogi and Sawhney, 1990a; Rastogi and Sawhney, 1990b). In addition, regeneration of tobacco plant from a cell line impaired in PA metabolism produced flowers with a second row of petals in place of anthers (Malmberg, 1980), while a MGBG-[methylglyoxal-bis (guanylhydrazone)]-resistant tobacco cell line with elevated PA levels also exhibited abnormal floral phenotype producing flowers with anthers in place of ovules (Malmberg and McIndoo, 1983). MGBG is a known inhibitor of S-adenosyl methionine decarboxylase (SAMdc), a critical enzyme in PA biosynthesis pathway. Inhibition of SAMdc by MGBG led to impaired flowering, which was reversed by Spd in *Spirodela punctata* (De Cantu and Kanderler, 1989). Similarly, inhibition of other PA-biosynthesis enzymes using alpha-difluoromethylarginine (DFMA), an arginine decarboxylase inhibitor, and alpha-difluoromethylornithine (DMFO), an ornithine decarboxylase inhibitor, prevented floral bud initiation and subsequent development of floral bud in tobacco cell culture explants (Tiburcio et al., 1988). Tobacco and petunia mutants impaired in PA production also exhibited abnormal flower phenotype (Malmberg and McIndoo, 1983, Gerats et al., 1988; Malmberg and McIndoo, 1988), whereas high levels of PA in a tomato mutant resulted in abnormal stamen development (Rastogi and Sawhney, 1990a). Although these studies provided indirect evidence in favor of a role of PAs in flower development, the differential role(s) of specific PAs in these processes has remained to be discerned.

To determine the role of Spd during plant development including flower initiation, maturation, fruit set, and seed set, we introduced a yeast spermidine synthase gene (*ySpdSyn*) under the control of either a fruit-ripening-specific promoter (*SlE8*) or a constitutive *CaMV35S* promoter in tomato (Nambeesan et al., 2010). The *ySpdSyn* transgenic line with fruit-specific promoter *SlE8* developed normal fruit and seed set, and the fruits had longer shelf life (Nambeesan et al., 2010). However, the expression of *ySpdSyn* under *CaMV35S* promoter resulted in two types of transgenic plant populations: one that normally set fruit and seed and the other that did not set fruit/seed and exhibited a range of flower and fruit phenotypes. Thus, these lines with constitutive expression of *ySpdSyn* were employed in our studies presented here to address the question of PA role in floral organ identity and fruit/seed set in tomato.

Our results demonstrate that flowers of the transgenic plants that set fruit and seeds exhibit significantly higher Spd/Put and Spd/Spm ratios compared to flowers from the transgenic plants that failed to set fruit and seeds. The lack of fruit and seed set was found associated with a reduction in the transcript levels of GA biosynthesis genes and *DELLA*, a negative regulator of GA signaling. We interpret these results to indicate that PAs influence floral organ identity and fruit set in tomato by modifying the genetic program that controls expression of GA biosynthesis and signaling genes.

## Materials and Methods

### Generation of Transgenic Plants and Phenotypic Measurements

Transgenic plants overexpressing yeast spermidine synthase (*ySpdSyn*) were generated as described previously (Nambeesan et al., 2010). The *ySpdSyn* gene (ScSpe3, systematic name: YPR069C) was amplified from a yeast genomic library and cloned in the sense orientation between a *CaMV35S* promoter and the 3’ end of a pea rbcS-E9 gene in pKYLX71 (Nambeesan et al., 2010). This construct was introduced into disarmed *Agrobacterium tumefaciens* LBA4404, and *Agrobacterium* strains harboring the chimeric constructs were used for transformation of tomato cv. Ohio 8245 cotyledons (Tieman et al., 1992). Successful plantlets obtained were transplanted into potted soil and grown in the greenhouse.

Fifteen independent transgenic plants expressing *CaMV35S-ySpdSyn* were generated. Two independent transgenic lines C13, C14 that exhibited floral morphological defects, impaired fruit and seed set, and sterility were propagated as vegetative cuttings and characterized alongside the isogenic wild-type (WT) Ohio 8245 control.

### Phenotypic Analysis of Floral Morphology

For phenotypic measurements, 27–36 flowers were harvested from WT and transgenic plants, and the number of sepals, petals, and stamens were counted. Any abnormality in the stamen morphology was noted. The flower size was measured diagonally across the flower. The flowers were dissected to observe the morphology of the gynoecium. Pollen morphology from dissected stamens was observed under a light microscope (Olympus, Center Valley, PA).

### Pollen Germination Assay

For pollen germination assay, open flowers were hydrated for 1 h in a Petri dish with wet Kim wipes to maintain a relative humidity close to 100%. The hydrated pollen from the flowers was spread on a glass slide with 30 µl germination medium [20 mM MES, 2% sucrose, 15% PEG 4000, 1 mM KNO_3_, 3 mM Ca(NO_3_)_2_, 0.8 mM MgSO_4_, and 1.6 mM H_3_BO_3,_ pH 6.0]. The pollen grains were incubated for 3 h in the dark at 25°C under high relative humidity by placing the glass slide on a moist filter paper in a Petri dish. Germination was terminated by the addition of 5 µl of phenol solution. Pollen germination was scored using a light microscope (Olympus, Center Valley, PA). Pollen tubes that were twice the diameter of the pollen grain were scored as positive for successful pollination. Pollen grains were examined from at least five flowers with 100–200 pollen counted per flower. Digital images were captured using the SPOT software (Draper, Utah).

### Polyamine Measurement

Mature leaf and tissue from various stages of flower development, including early buds (0.1–0.7 mm in length), late buds (0.8–1.5 cm in length), and fully open flowers were harvested. Three independent biological replicates at each developmental stage were collected and were frozen immediately in liquid nitrogen and stored at −80°C until analyzed. All samples were processed and quantified in triplicates for polyamine measurement as described previously (Nambeesan et al., 2010).

### Semiquantitative and Quantitative RT-PCR

Mature leaf and flowers were collected at two stages of development, ∼135 early buds (0.1–0.7 mm in length) and 75 late buds (0.8–1.5 cm in length) from WT, C13, and C14 lines. Total RNA was extracted, and cDNA synthesis was performed following the manufacture’s recommendation (Promega, Madison, WI). Briefly, total RNA (1 μg) was treated with DNase and ImPromII reverse transcriptase to perform reverse transcription. The cDNA was diluted five times, and 1 μl of diluted cDNA was used for semiquantitative reverse transcription PCR (RT-PCR) in a 10-μl total reaction volume. For each primer pair, cycle number was determined for exponential amplification, and all subsequent reactions were performed for the predetermined number of cycles. Each PCR reaction typically included the following cycles: 95°C (3 min); [95°C (30 s); 55/56°C (30 s); 70°C (1 min)] 25 cycles; 72°C, 10 min. Tomato *ACTIN* gene was used for normalization. After PCR amplification, the products were separated on a 1.2% agarose gel, stained with 0.5 μg/ml ethidium bromide, and images were captured and processed using Image J software (National Institutes of Health, Bethesda, Maryland). Primers used for PCR amplification are listed in **Supplemental Table 1**.

Quantitative RT-PCR analysis was performed using real-time PCR (Stratagene Mx3005P, San Diego, CA). All primers (**Supplemental Table 1**) along with control *ACTIN* (*SlACTIN*) were validated for relative gene expression analysis. One microliter of the cDNA was utilized in a 15-μl reaction using the SYBR green-PCR master mix (Applied Biosystems, Foster City, CA). Melting curve analysis was performed after the PCR reaction to determine specificity of the PCR products. At least three biological replicates were analyzed for gene expression analyses. Statistical analyses were performed using one-way analysis of variance to compare across transgenic C13 and C14 lines and WT separately for a given developmental timepoint or treatment, using JMP Pro 12 (SAS Institute, Cary, NC, USA). Means were separated using Tukey’s honestly significant difference (HSD) test (α = 0.05).

## Results

### Fruit and Seed Set in *ySpdSyn* Transgenic Tomato Lines

Eleven independent events in transgenic tomato lines positive for the expression of *CaMV35S‐ySpdSyn* were evaluated in greenhouse. Five of them—C5, C11, C12, C13, and C14—were found severely impaired in fruit set. They varied from having no seeds to very few seeds and exhibited partial parthenocarpy. Other four independent transgenic lines—C1, C3, C4, and C15—exhibited normal fruit set, but their seed number/fruit varied being 18, 15, and 30% lower in C1, C3, and C4, respectively, except for C15 in which seed numbers were similar to the WT control (**Table 1**). Variable phenotypes, including impaired fruit and seed set and seed germination among the independent transgenic plants, may have resulted from the influence of transgene integration site(s) (De Buck et al., 2013).

**Table 1:** Effect of overexpression of *ySpdSyn* on seed number.

Genotype	Number of seeds/fruit	% WT
WT	935	
C1	770	82
C3	792	85
C4	653	70
C15	920	98

Seeds from 10 fruits were extracted for each genotype and counted. Shown are the average 10 independent extraction of seeds from each genotype.

### Expression of *ySpdSyn* and PA Quantification in C13 and C14 Transgenic Lines

**Figure 1:**
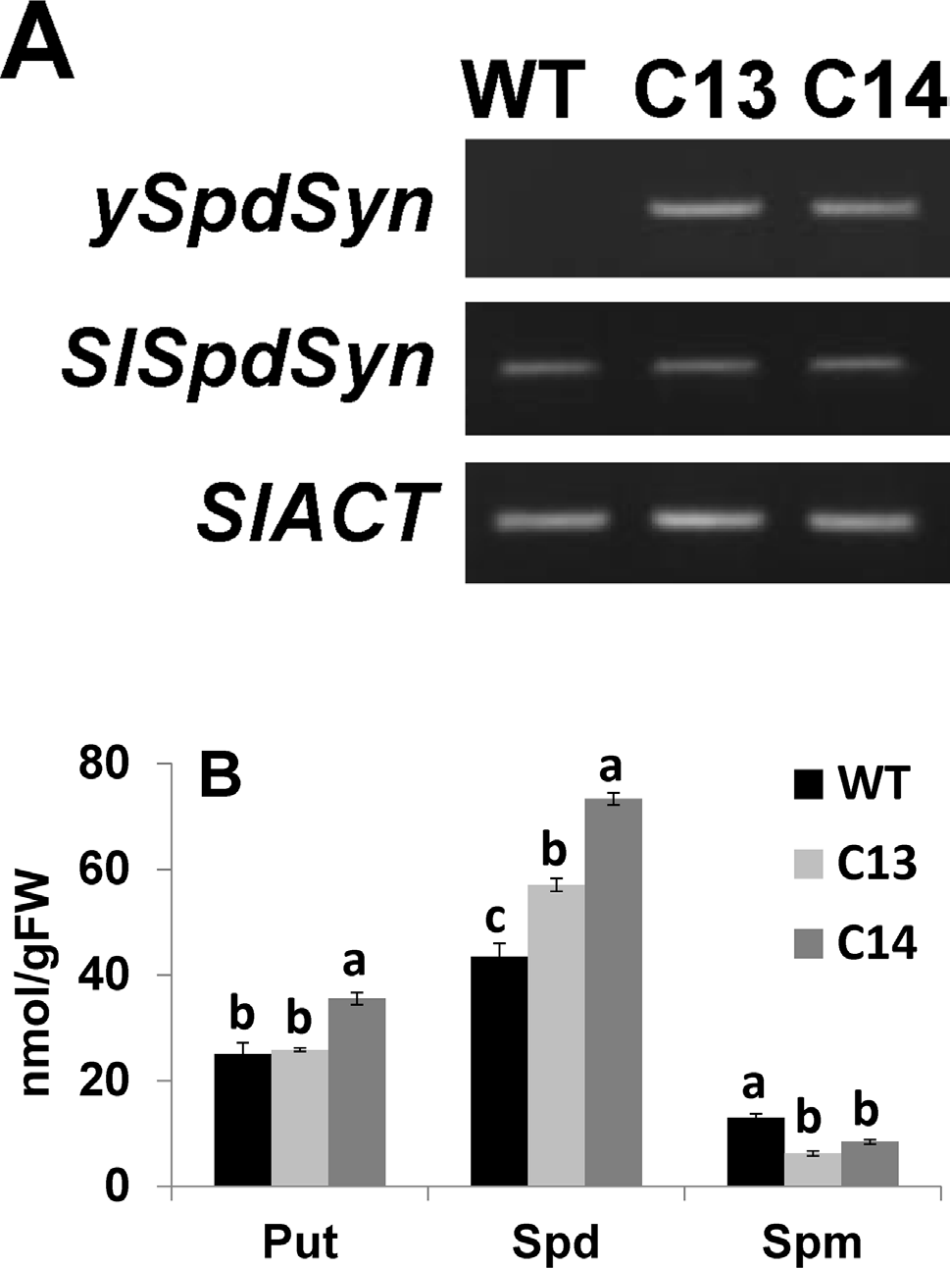
Generation and characterization of transgenic plants overexpressing yeast spermidine synthase (*ySpdSyn*). **(A)** The *ySpdSyn* construct was cloned under the *CaMV35S* promoter in pKYLX71 vector. Shown are levels of endogenous *SlSpdSyn* and the control *SlACT* using semiquantitative RT-PCR analysis indicating the expression of *ySpdSyn* and endogenous tomato *SlSpdSyn* in WT, C13, and C14 leaves. The tomato *Actin* gene was used as an internal control (*SlACT*). **(B)** Polyamine levels in WT, C13, and C14 leaves measured using HPLC. Shown are mean values and standard from three independent biological leaf replicates. Means followed by the same letter are not significantly different from each other based on one-way analysis of variance (α = 0.05) for a given polyamine (PA). Put, putrescine, Spd, spermidine; and Spm, spermine.

Expression of *ySpdSyn* and endogenous *SlSpdSyn* in tomato leaves of C13 and C14 T_0_ lines is shown in **Figure 1A**. Both transgenic lines expressed *ySpdSyn* and *SlSpdSyn*, while, as expected, WT leaves expressed only the endogenous *SlSpdSyn* gene. The *SlSpdSyn* transcript levels remained unaltered in C13 and C14 lines compared to WT leaves, which indicated lack of endogenous gene silencing (**Figure 1A**). Quantification of different polyamines in these leaves indicated that Spd levels increased by 1.3- and 1.7-fold in C13 and C14 lines, respectively, as compared to the WT (**Figure 1B**). However, the transgenic event caused a 2.1- and 1.5-fold decrease in Spm in C13 and C14, respectively, compared to WT plants. Leaves from C14 lines exhibited significant 1.4-fold increase in Put compared to WT leaves, whereas C13 leaves did not show any change in Put levels compared to WT leaves (**Figure 1B**).

### Altered Floral Morphology of *ySpdSyn* Under *CaMV35S* Transgenic Plants

**Figure 2:**
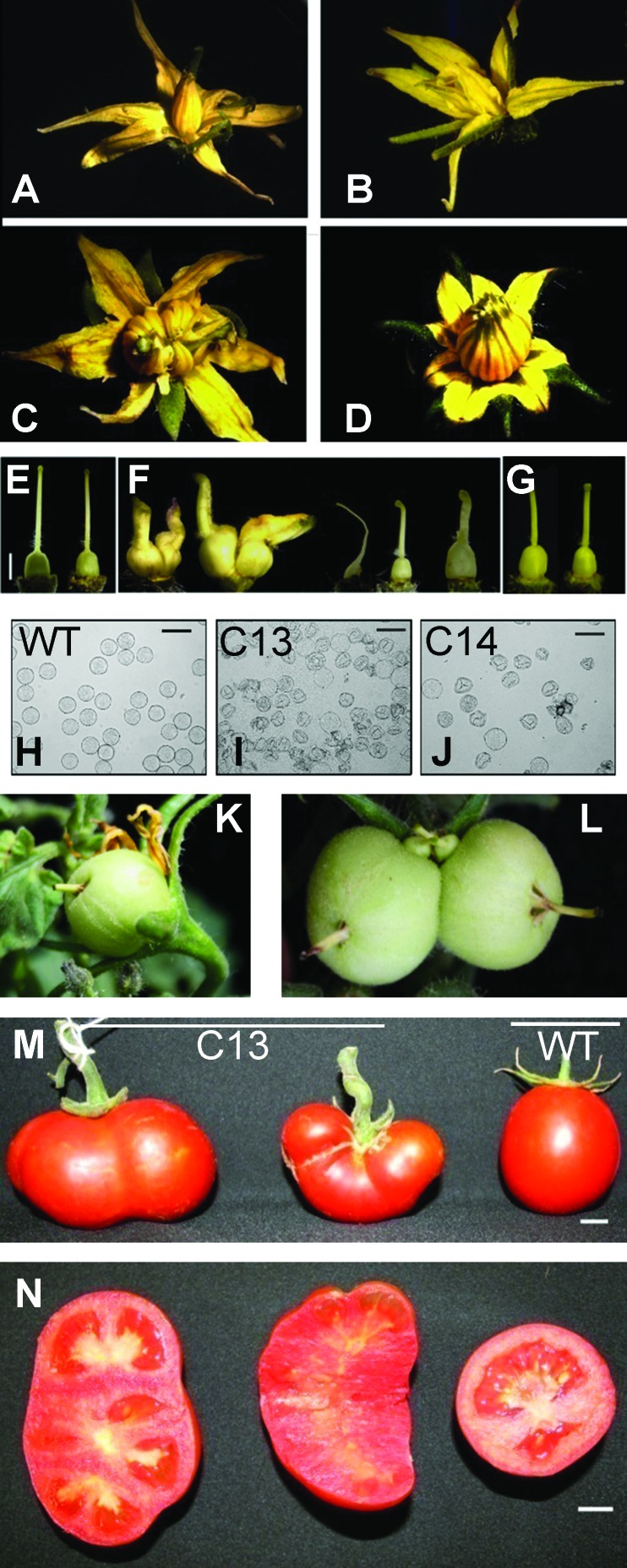
Altered floral and fruit characteristics in lines overexpressing *ySpdSyn*. **(A)** Wild-type flower. **(B)** Impaired staminal cone formation in C14. **(C)** Higher stamen number with impaired staminal cone in C13. **(D)** Higher stamen number and petal number in C13. **(E**–**G)** Alterations in carpel development in C13 **(F)** and C14 **(G)** in comparison to the wild type **(E)**. **(H**–**J)** Changes in pollen morphology of C13 **(I)**, C14 **(J)** in comparison to wild type **(H)**. **(K)** Delayed petal abscission in C13. **(L)** Multiple fruit formation from a single flower in C13. **(M**, **N)**. Altered fruit shape and multilocular fruits in C13 in comparison to wild-type fruit.

The floral and fruit morphology in transgenic and WT plants was evaluated to determine the basis of impaired fruit and seed set in C13 and C14 lines. Flower size or floral organ number did not differ significantly among these lines compared to WT plants. All genotypes had five sepals per flower. However, the average petal number was significantly different in line C13 (6.6 petal/flower) as compared to WT and C14 which had 5.8 and 5.3 petals/flower, respectively (**Figure 2D**). Similarly, the average number of stamens in C13 (7 per flower) was significantly higher than the WT and C14 lines that had 5.8 and 5.4 stamens per flower, respectively (**Figure 2D**). However, C13 line had severe morphological defects, with ∼17% flowers having 8–10 stamens in comparison to 5.4 in the WT. Most flowers from C14 and C13, however, did not form a normal staminal cone (**Figure 2A–C**). Dissection of flowers from transgenic lines revealed that carpels from C13 (**Figure 2F**) and C14 flowers (**Figure 2G**) were significantly enlarged as compared to the WT flowers (**Figure 2E**). Occasionally, fusion of multiple gynoecia at the base was observed in C13 flowers. The length of the style was reduced in C13 and C14 flowers (**Figures 2E–G**) with the tip of the style close to the stigmatic surface curved in transgenic flowers (**Figures 2E–G**). Other phenotypes in transgenic flowers included delayed abscission of petals during fruit growth (**Figure 2K**), multiple fruit set in the same flower by fusion of multiple gynoecia at the base (**Figures 2L, M**), and altered fruit shape with multiple locules (**Figure 2N**).

We next investigated pollen morphology and germination to determine the basis of differences in fruit set in transgenic lines. WT pollen appeared spherical and intact, and 96% of the pollen displayed a normal morphology (**Figure 2H**). However, ∼68% of pollen from C13 and 37% from C14 flowers exhibited a collapsed morphology (**Figures 2I, J**). *In vitro* pollen germination assays indicated reduced pollen germination in C13 and C14 lines compared with WT. Among the intact-appearing pollen, 79% of WT and only 17% of C13 and 40% of C14 pollen grains germinated (data not shown). None of the abnormal looking pollen grains germinated (data not shown). The reciprocal crosses using C13 and C14 pollen on WT stigma did not result in fruit set, but pollination of C13 and C14 using WT pollen resulted in 100 and 80% parthenocarpic fruit set, respectively (data not shown), Among the fruit that set seed in C14, only one seed was present. These results indicated that fruit development occurred without fertilization. Reduced seed set in C14 indicated partial female sterility.

### PA Homeostasis and Changes in Spd/Put and Spd/Spm Ratios

**Figure 3:**
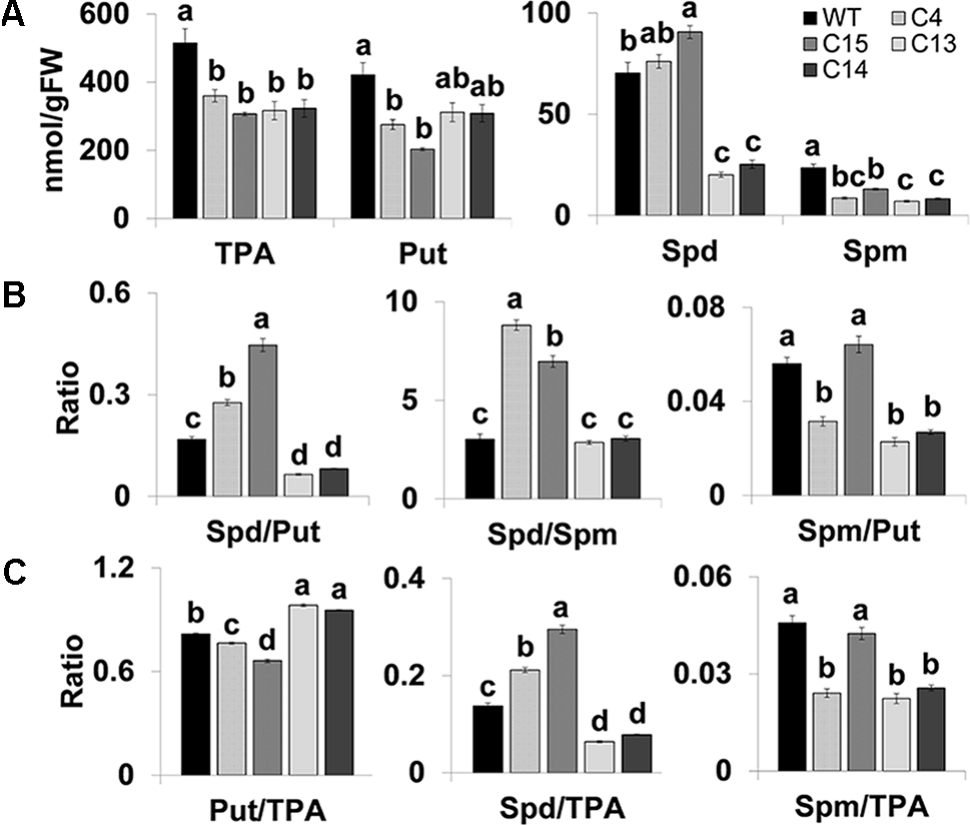
Changes in the free total PAs (TPA), Put, Spd, and Spm **(A)**, ratios of Spd/Put, Spd/Spm, and Spm/Put **(B)**, and ratios of Put, Spd, and Spm to TPA **(C)** in fully open flowers of WT, C4, C15, C13, and C14 plants. WT, Wild-type Ohio 8245; C4, C15, C13, and C14 transgenic plants harboring *CaMV-SpdSyn* transgene. C4 and C15 plants displayed fruit and seed set, while the C13 and C14 plants exhibited abnormal flower phenotype and impaired seed set. Shown are the mean values and standard errors from the three independent biological replicates. Means followed by the different letters are significantly different from each other based on one-way analysis of variance (α = 0.05) for a given polyamine (PA).

We determined the levels of free polyamines (Put, Spd, and Spm) in fully opened flowers from WT and four *CaMV-ySpdSyn* lines to evaluate if altered PAs homeostasis was associated with the observed phenotypes (**Figure 3**). The steady-state Spd level in the flowers of other transgenic lines, viz., C4, C15, and WT plants was similar; these genotypes had normal fruit and seed set (**Figure 3**). The flowers of C13 and C14 lines exhibited parthenocarpic phenotype and had significantly lower Spd levels than the WT flowers (**Figures 2** and **3**). Notably, the Spd/Spm ratio in the flowers of C4 and C15 plants was 8.8 and 7.0, respectively, and only 2.9 and 3.1 in C13 and C14 flowers, respectively (**Figure 3**). Similarly, the Spd/Put ratio was significantly higher in C4 and C15 flowers compared to the WT flowers but significantly lower in C13 to C14 flowers. This pattern was also reflected in the Spd/TPA (**Figure 3**). Notably, the ratio of Put/TPA was significantly lower in C4 and C15 flowers and significantly higher in C13 and C14 flowers compared to WT flowers, respectively. Consistent patterns for Spm/TPA ratio were not observed for transgenic flowers from the four independent lines examined (**Figure 3**). Taken together, these results suggest that reduced Spd and Spd/Put ratios are associated with the flower phenotype and may therefore affect fruit and seed set in tomato.

### Establishment of Polyamine Homeostasis During the Early Flower Development

**Figure 4:**
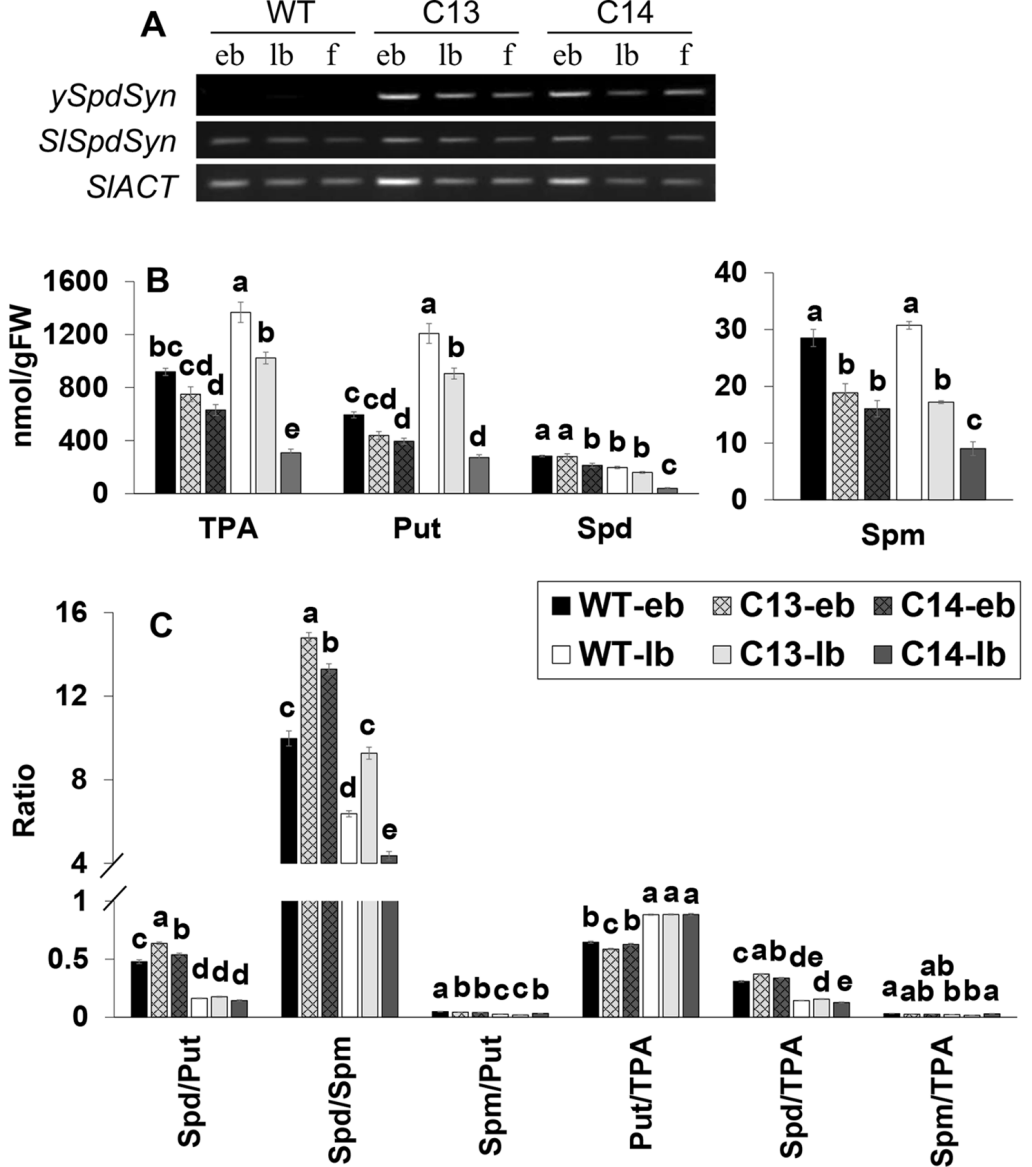
Expression of *ySpdSyn* and *SlSpdSyn* genes **(A)**, changes in the levels of free and total PAs **(B)**, and changes in their ratios **(C)** during early and late stages of flower bud development in WT, C13, and C14 lines. Tomato *ACTIN* was used as a reference (*SlACTIN*). Flower development stages include early buds (eb; 0.1–0.7 cm) and late buds (lb; 0.8–1.5 cm). PA levels were measured in three independent biological replicates. Other details are the same as in **Figure 3**. Means followed by the different letters are significantly different from each other based on one-way analysis of variance (α = 0.05) for a given polyamine (PA).

Expression patterns of *ySpdSyn* and endogenous *SlSpdSyn* were compared at various stages of flower development including early and late bud stages and fully open flowers. The *ySpdSyn* transcripts were present at all the stages of flower development in the C13 and C14 lines and, as expected, not in the WT (**Figure 4A**). As anticipated, *SlSpdSyn* transcript was present at all the stages of flower development, indicating the lack of silencing of the endogenous or *ySpdSyn* transgene in the transgenic lines (**Figure 4A**).

We quantified the levels of Put, Spd, and Spm in early and late flower buds to determine if the observed changes in various PA levels described in fully opened WT flower were also present during the flower development in C13 and C14 genotypes that had abnormal fruit and seed set (**Figure 4B**). The Spd level was not significantly different at early and late bud stages in the C13 line but was significantly lower in the C14 line at both stages of bud development. Furthermore, Spm levels in the C13 and C14 genotypes were significantly lower than the WT at early and late bud stages of flower development, whereas TPA and Put levels were lower at the latter stage.

The Spd/Put ratio was significantly higher at early bud stage in the C13 and C14 lines but not at late bud stage as compared with the WT. Spd/Spm ratio was significantly higher in the early and late bud stage in C13, whereas it decreased at the late bud stage in C14 line (**Figure 4C**). A significant decrease in Spm/Put ratio in both the C13 and C14 genotypes compared to WT was apparent only at the early bud development stage (**Figure 4**). Although the ratio of Spd/TPA was higher at the early bud stage, however, in general, Put/TPA and Spm/TPA ratios in both genotypes at the early and late bud stages of development were not different from the WT.

### Steady-State Levels of *SlDELLA* and GA Biosynthesis Genes Are Lower in the Flowers of *ySpdSyn* Transgenic Plants With Impaired Fruit and Seed Set

**Figure 5:**
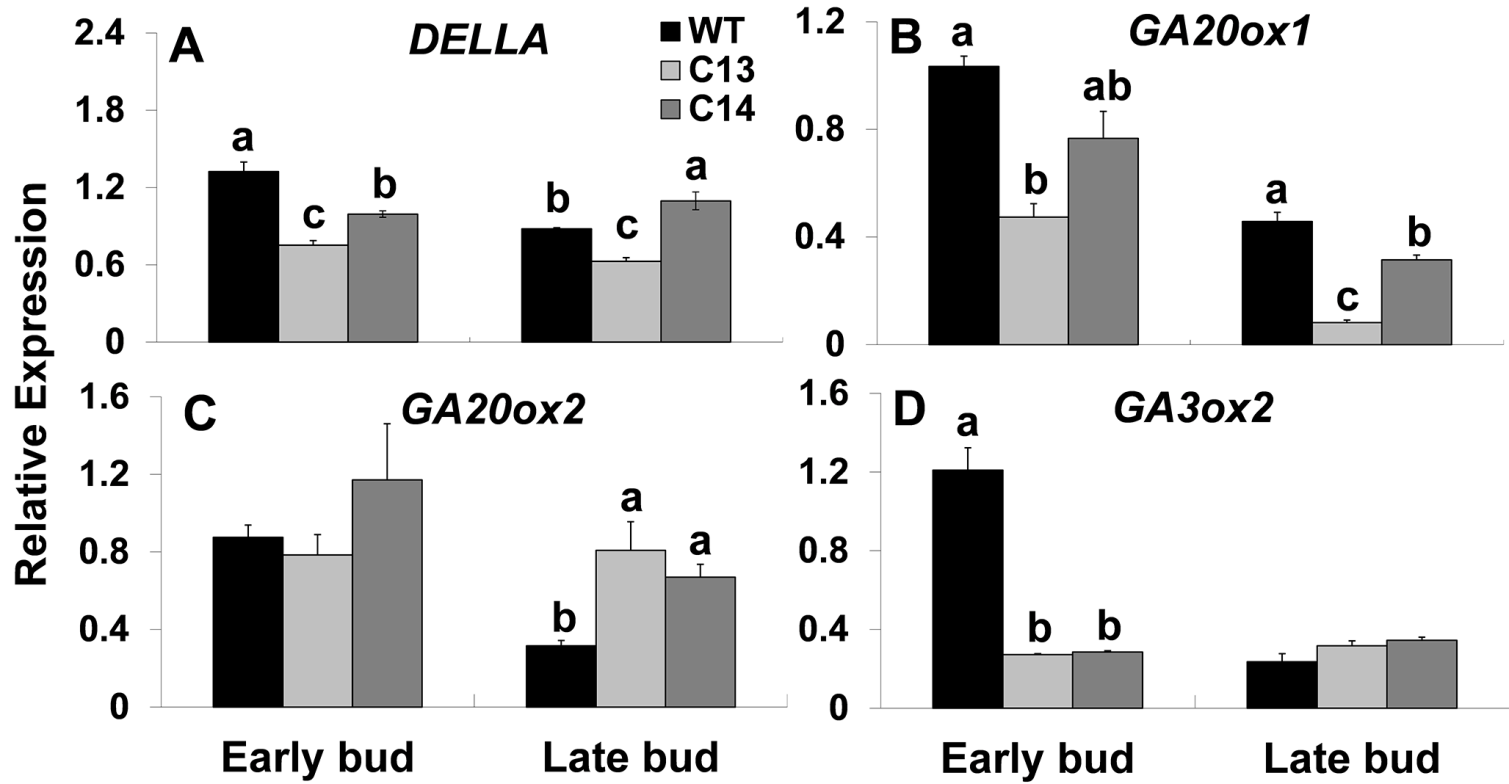
Transcript levels of *SlDELLA* and GA-biosynthesis genes during flower development of *ySpdSyn and WT* tomato lines. Flower development stages, early buds (0.1–0.7 cm) and late buds (0.8–1.5 cm), were harvested from plants grown in a green house. Expression of the tomato homologue of **(A)** *DELLA* (*SlDELLA*); **(B)** GA-20oxidase-1 (*GA20ox1*); **(C)** GA-20oxidase-2 (*GA20ox2*); and **(D)** GA-3oxidase-2 (*GA3ox2*), during flower development in wild type, C13 and C14 flower buds was analyzed using quantitative RT-PCR (Supplemental File 1). The relative gene expression analysis was performed by the ∆∆Ct method using tomato *ACTIN* as a reference gene. Data shown are mean + standard error based on ting three independent biological replicates of WT, C13, and C14 at early and late bud stage of flower development. Means followed by the same letter are not significantly different from each other based on one-way analysis of variance (α = 0.05) for a given tissue type of a gene.

Flower initiation and development are known to be regulated by the hormone gibberellins (GAs) (Davis, 2009). Therefore, we surmised that PAs and GAs may interact to regulate floral programs. To understand such an association between GAs and PAs, we analyzed the expression of tomato *DELLA* (*SlDELLA*) and GA-biosynthesis genes (**Figure 5**). *DELLA* expression decreased significantly at early stages of bud development in C13 and C14 lines being 1.8- and 1.4-fold lower in C13 and C14, respectively, as compared to WT (**Figure 5A**).

The expression of *GA20-oxidase1* (*GA20ox1*) decreased by 2.2-fold in the C13 line at the early bud stage, decreasing further by 5.6-fold in the late bud stage (**Figure 5B**). A similar pattern was obtained for flower bud development in C14 line. Expression of *GA20-oxidase2* (*GA20ox2*) was not significantly different at the early bud stage of both the transgenic lines; however, at the late bud stage, *GA20ox2* expression was elevated ∼2- and ∼2.5-fold in the C13 and C14 lines, respectively (**Figure 5C**). In contrast, *GA3ox2* expression was significantly downregulated ∼4-fold in both C13 and C14 lines at the early bud stage with no significant differences at the late bud stage between the WT and two transgenic lines (**Figure 5D**).

## Discussion

Put, Spd, and Spm have two, three, and four positive charges, respectively, and thus can compete differentially to bind various biomolecules (Igarashi and Kashiwagi, 2010). Changes in the levels of any specific PAs (Put, Spd, or Spm) may affect their binding to biomolecules resulting in the perturbation of various biological processes, including transcription and metabolome (Mattoo et al., 2006; Srivastava et al., 2007). We demonstrate here that the expression of *CaMV35S-ySpdSyn* plants results in significant changes in the ratios of Spd/Put, Put/TPA, and Spd/TPA in transgenic flowers. Transgenic plants expressing *ySpdSyn* gene under the control of *CaMV35S* promoter gave rise to two distinct populations of transgenic plants. About one-half of the independent transgenic plants exhibited normal flower development with fruit and seed set, while the other half of the transgenic population showed floral abnormalities and parthenocarpic fruit set (**Figure 2**). The Spd level in the transgenic flower with normal fruit and seed set phenotype was similar to WT flowers but lower by threefold in transgenic flowers with abnormal parthenocarpic fruit than WT flowers. We propose that such alterations of individual PAs and their ratios affect the phenotypes observed in transgenic flowers. Altered PA homeostasis with increased PA ratios such as Spd to Spm ratio has been recently reported in Snyder–Robinson syndrome (Murray-Stewart et al., 2018). These findings provide further support and add to the role(s) of PAs in flower development and parthenocarpy (Rastogi and Sawhney, 1990b; Antognoni et al., 2002; Fos et al., 2003; Sinha and Rajam, 2013; Chen et al., 2014)

Furthermore, these data corroborate earlier envisaged reports that each polyamine Put, Spd, and Spm differentially influence plant phenotypes, metabolism, and gene expression (Handa and Mattoo, 2010; Mattoo et al., 2010; Tiburcio and Alcázar, 2018). Thus, Spd and Spm were positively correlated with tomato fruit-quality attributes and specific metabolites in poplar cell culture and tomato pericarp in contrast to Put, which exhibited a negative correlation with these same traits (Handa and Mattoo, 2010; Mattoo et al., 2010). The importance of PA ratios in regulating various biological systems including plants and mammalian systems has been previously suggested, but their physiological significance has remained elusive (Huang et al., 2004; Murray-Stewart et al., 2018). Changes in PA ratios were also found in Gy mice and human patients with Snyder–Robinson syndrome, a genetic condition, resulting from the lack of SpmSyn activity. Both these phenotypes were found associated with increased Spd/Spm ratio due to a decrease in Spm (Pegg, 2016). Since high levels of Spd restored the normal growth of Gy mice lacking SpdSyn in cultured fibroblast, it has also been suggested that the Spm/Spd ratio plays an important role in myocyte differentiation (Luchessi et al., 2009). Interestingly, the Spd/Spm ratio has also been implicated in altering activity of Kir channels affecting the resting membrane potential, cardiac and neuronal electrical activity, and electrolyte balance (Stanfield and Sutcliffe, 2003). In addition, our findings are in sync with the contention that cellular levels of individual PAs, their homeostasis, particularly ratio among different PAs, regulate various physiological responses (Murray-Stewart et al., 2018).

Molecular basis of reduced levels of Put, Spm, and total PAs in transgenic flowers from all the transgenic lines tested here and reduction in Spd levels in only about half the independent transgenic lines tested here is not clear. Variable expression of the transgenes under the *CaMV35S* promoter has been previously observed in several investigations (Tieman et al., 1992; Somssich, 2019). Nonetheless, that the ratio of Spd/Spm and Spd/Put in transgenic flower with normal fruit and seed set were higher compared to transgenic abnormal transgenic flowers (**Figure 3**) may indicate that it is Spd ratio with Put and Spm that is crucial for the observed flower phenotypes. This warrants further investigation.

PA and GA metabolism and signaling were linked to floral and early fruit development in tomato, but the nature of these interactions was not clear (Anwar et al., 2015). High GA levels in *pat-2* ovaries were associated with increased PA biosynthesis and higher Spm levels (Fos et al., 2003). In *Arabidopsis*, GA promotes flower development by suppressing expression of DELLA group of proteins and, in turn, promotes the expression of floral homeotic genes (Okamuro et al., 1997; Cheng et al., 2004; Tyler et al., 2004; Yu et al., 2004; Cao et al., 2006). The tomato *procera* mutant in which *SlDELLA* gene is mutated has a constitutively induced GA phenotype with facultative parthenocarpy and meristematic alterations (Carrera et al., 2012). In addition, overexpression of *GA20oxidase* resulted in some flowers having protruding stigma and partial parthenocarpy in tomato (García-Hurtado et al., 2012). In the present study, the expression of *SlDELLA* and GA-biosynthesis-related genes, *GA20ox1* and *GA3ox2*, was significantly downregulated in those transgenic lines that had abnormal flower phenotype. Furthermore, *DELLA*-silenced lines are downregulated in *GA20ox* and *GA3ox* during early fruit development, suggesting that silencing of *DELLA* may result in higher GA levels which in turn reduce the expression of GA-biosynthetic genes through a feedback regulation (Martí et al., 2007). For instance, GA-deficient *Arabidopsis* mutants have a higher expression of GA biosynthesis genes, *AtGA20ox1* and *AtGA3ox1*, which could be reduced by GA application (Yamaguchi et al., 1998; Xu et al., 1999; Matsushita et al., 2007). In light of these observations, our data on decreased expression of *GA20ox1* and *GA3ox2* in the C13 and C14 transgenic lines may be a result of a similar feedback regulation and suggest that PAs play an important role in flower development and fruit set likely by interacting with GA signal transduction and biosynthetic pathways in tomato. The contention that Spd levels may have an important role in flower development has been previously proposed (Malmberg, 1980; Malmberg and McIndoo, 1983; Gerats et al., 1988; Malmberg and McIndoo, 1988; Tiburcio et al., 1988; Galston and Sawhney, 1990; Rastogi and Sawhney, 1990a, Rastogi and Sawhney, 1990b; Pritsa and Voyiatzis, 2004, Pritsa and Voyiatzis, 2005; Alburquerque et al., 2006; DeDios et al., 2006). However, much more definitive evidence is required to conclude that altered PA ratios regulate floral developmental process(es). In conclusion, our studies present a compelling suggestion for a nexus between PA ratios and regulation of developmental programs in plants.

## Data Availability Statement

All datasets for this study are included in the manuscript/**Supplementary files**.

## Author Contributions

All authors had equal roles in conceptualization, data analyses, and manuscript preparation.

## Funding

Studies were partly supported by a US–Israel Binational Agricultural Research and Development Fund to AH and AM (grant no. IS-3441-03) and USDA-IFAFS program grant (award no. 741740) and USDA/NIFA Hatch IND011872 to AH. AM is supported through USDA-ARS intramural Project No. 8042-21000-143-00D.

## Conflict of Interest

The authors declare that the research was conducted in the absence of any commercial or financial relationships that could be construed as a potential conflict of interest.
