# Supplementary material for: Nexus Between Spermidine and Floral Organ Identity and Fruit/Seed Set in Tomato
Source: Front Plant Sci. 2019 Sep 25;10:1033. doi: 10.3389/fpls.2019.01033 (PMC6774279; doi:10.3389/fpls.2019.01033)
Supplement: Supplementary file 1 [file Table_1.docx]

**Supplemental Information**

**Nexus Between Spermidine and Floral Organ Identity and Fruit/Seed Set in Tomato**

Savithri U. Nambeesan^1^, Autar K Mattoo^2^ and Avtar K Handa^3*^

^1^Department of Horticulture, University of Georgia, Athens, GA, USA; ^2^Sustainable Agricultural Systems Laboratory, USDA-ARS, Beltsville Agricultural Research Center, Beltsville, MD, USA; ^3^Center of Plant Biology, Department of Horticulture and Landscape Architecture, Purdue University, West Lafayette, IN, USA.

**Supplemental Table 1**: List of primers used for Real-time and Semi-quantitative RT-PCR

| Name | Sequence |
| --- | --- |
| GA20ox1F | CCCGATTTCACATGGCCTACTCTTC |
| GA20ox1R | AGCTTGTGTAGTAGTGTTGTGTTGATCA |
| GA20ox2F | CCATCCTCCATCAAGACAACGTCTC |
| GA20ox2R | AGGATTATTAGAGTCCACCAATTCTGCC |
| GA20ox3F | CAAGTTTTTGTGGATAATGAGTGGCACA |
| GA20ox3R | TGTTAGAATCCACTAATTCATTTGGTGGAG |
| GA3ox-1F | GACCATAGGCACCCACCCT |
| GA3ox-1R | GTACCCCTTTATGATCTTTGGCATCAGT |
| GA3ox-2F | TGGTAGACCAAAGGAACCCTCAAA |
| GA3ox-2R | GCCACTTTGATCCTTGGAATTGGC |
| DellaF | TGGGTCTTCGTCTTCAGCTT |
| DellaR | GAACGCATTTGAACCCAGAT |
| TM4F | CAGCTTGTTCCTACTGATCATACCTCC |
| TM4R | GTTGTTTTGCTCCTGCAATGCTCTG |
| TM5F | CCGAATATATCAACACGAGAAGCACTGG |
| TM5R | CTGTTGGCTTCGTTCAATGCATGT |
| TDR6F | GACACCTGCAAGAAGAAGGC |
| TDR6R | ACAGCAGAGTGGTAATGCCC |
| TM29F | CACCAGGACACAAATGATGC |
| TM29R | TTGAGGATGTTGCTGCTGAC |
| TAGF | GGATCGAAAACACGACGAAT |
| TAGR | CCTCTCGATTGTTGCTTTCA |
| LeActinF | TGGTCGTACCACCGGTATTGTG |
| LeActinR | AATGGCATGTGGAAGGGCATAC |
| SQRT SlSpdSynF | GCAGAAACAGAGATGGAGGC |
| SQRT SlSpdSynR | CCAATGTGGAGATTCACACG |
| SQRT ySpdSynF | ACTCACCCAACTATTGTAGACGGC |
| SQRT ySpdSynR | CACGTAGTGGCTTCTTGACATTGC |
| SQRT LeActF | ATGTATGTTGCCATCCAGGCTG |
| SQRT LeActR | CCTTGCTCATCCTATCAGCAGCAATACC |
